# Quantitative Assessment of Levonorgestrel Binding Partner Interplay and Drug‐Drug Interactions Using Physiologically Based Pharmacokinetic Modeling

**DOI:** 10.1002/psp4.12572

**Published:** 2020-12-13

**Authors:** Brian Cicali, Karthik Lingineni, Rodrigo Cristofoletti, Thomas Wendl, Joachim Hoechel, Herbert Wiesinger, Ayyappa Chaturvedula, Valvanera Vozmediano, Stephan Schmidt

**Affiliations:** ^1^ Center for Pharmacometrics and Systems Pharmacology Department of Pharmaceutics College of Pharmacy University of Florida Orlando Florida USA; ^2^ Bayer AG Pharmaceuticals Leverkusen Germany; ^3^ Department of Pharmacotherapy System College of Pharmacy University of Northern Texas Health Science Center Fort Worth Texas USA

## Abstract

Levonorgestrel (LNG) is the active moiety in many hormonal contraceptive formulations. It is typically coformulated with ethinyl estradiol (EE) to decrease intermenstrual bleeding. Due to its widespread use and CYP3A4‐mediated metabolism, there is concern regarding drug‐drug interactions (DDIs), particularly a suboptimal LNG exposure when co‐administered with CYP3A4 inducers, potentially leading to unintended pregnancies. The goal of this analysis was to determine the impact of DDIs on the systemic exposure of LNG. To this end, we developed and verified a physiologically‐based pharmacokinetic (PBPK) model for LNG in PK‐Sim (version 8.0) accounting for the impact of EE and body mass index (BMI) on LNG’s binding to sex‐hormone binding globulin. Model parameters were optimized following intravenous and oral administration of 0.09 mg LNG. The combined LNG‐EE PBPK model was verified regarding CYP3A4‐mediated interaction by comparing to published clinical DDI study data with carbamazepine, rifampicin, and efavirenz (CYP3A4 inducers). Once verified, the model was applied to predict systemic LNG exposure in normal BMI and obese women (BMI ≥ 30 kg/m^2^) with and without co‐administration of itraconazole (competitive CYP3A4 inhibitor) and clarithromycin (mechanism‐based CYP3A4 inhibitor). Total and free LNG exposures, when co‐administered with EE, decreased 2‐fold in the presence of rifampin, whereas they increased 1.5‐fold in the presence of itraconazole. Although changes in total and unbound exposure were decreased in obese women compared with normal BMI women, the relative impact of DDIs on LNG exposure was similar between both groups.

**Study Highlights**

**WHAT IS THE CURRENT KNOWLEDGE ON THE TOPIC?**

☑ Levonorgestrel (LNG) is a progestin‐based hormonal contraceptive that is, either alone or in combination with ethinylestradiol (EE), widely used around the world. It is partially cleared by CYP3A4, which makes it subject to drug‐drug interactions (DDIs). DDIs with CYP3A4 inducers have been associated with unintended pregnancies. Yet, the extent is currently unclear.

**WHAT QUESTION DID THIS STUDY ADDRESS?**

☑ What is the impact of DDIs on free and total LNG exposure when given alone or in combination with EE?

**WHAT DOES THIS STUDY ADD TO OUR KNOWLEDGE?**

☑ Both total and unbound LNG exposures decreased ~ 2‐fold in the presence strong CYP3A4 inducers and increased ~ 1.5‐fold in the presence of strong CYP3A4 inhibitors. Although the magnitude of exposure was decreased in obese populations compared with normal body mass index, the relative impact of DDIs remained similar between both groups.

**HOW MIGHT THIS CHANGE DRUG DISCOVERY, DEVELOPMENT, AND/OR THERAPEUTICS?**

☑ This study sets the stage for the establishment of a general framework for evaluating the impact of DDIs on the efficacy and safety of hormonal contraceptives. The results of this effort will support the refinement of current drug class labels and help risk stratify individual agents. Furthermore, future work will allow for the identification of minimum effective exposure thresholds to support novel formulation development.

Levonorgestrel (LNG) is a synthetic progestogen present in many progestin‐based hormonal contraceptives. LNG formulations include progestin‐only pills, subdermal implants, intrauterine devices, combined hormonal contraceptives (CHCs), and emergency contraception.^1^ Hormonal contraceptive agents are widely used throughout the world, mainly to prevent unintended pregnancies, with higher use rates in developed countries compared with developing countries. LNG exerts its effect via multipronged mechanisms‐of‐action, including inhibition of ovulation, thickening of cervical mucus to inhibit sperm penetration, and desynchronization of the endometrial changes necessary for implantation.^2^ Oral progestins are typically combined with an estrogen, such as ethinyl estradiol (EE), to decrease intermenstrual bleeding and to further decrease ovulation.^3^ Phase III clinical trial results suggest that CHCs have perfect‐use failure rates of 1–2%.^4^ However, failure rates in the general population are higher and amount to ~ 9%, possibly due to nonadherence or drug‐drug interactions (DDIs).^5^

LNG and other hormonal contraceptives are cleared by cytochrome P450‐3A4 (CYP3A4) as well as phase II enzymes, including uridine 5'‐diphospho‐glucuronosyltransferase or sulfotransferases.^6^ Many medications concomitantly administered with CHCs can inhibit or induce these enzymes to increase or decrease LNG exposure. Specifically, co‐administration of CYP3A4 inducers has been associated with an elevated risk of unintended pregnancies due to decreased LNG exposure.^7^, ^8^, ^9^, ^10^ The magnitude of these DDIs is frequently unclear because hormonal contraceptives are typically old(er) compounds for which no dedicated DDI studies have been conducted. Therefore, the US Food and Drug Administration (FDA) convened a workshop in November 2015 to seek input from key opinion leaders on the use of CHCs and interacting drugs.^11^ The FDA subsequently published a draft guidance for industry on labeling for CHCs in 2017.^12^ Section 7 of this draft guidance is subject to DDIs. It contains a drug‐class label for CHCs highlighting the importance of CYP3A4 inducers relative to CYP3A4 inhibitors and clinical strategies to minimize the impact of DDIs on the efficacy/safety of CHCs. Whether or not this drug class label can be generalized to all CHCs remains unclear. Besides interactions at the enzyme level, pharmacokinetics (PKs) of LNG in CHC formulations can be influenced by EE resulting in a dynamic change in the plasma concentrations of sex‐hormone binding globulin (SHBG), LNG’s high‐affinity binding partner.^13^ The situation becomes more complex because SHBG plasma concentrations are impacted by changes in intrinsic factors, such as body mass index (BMI).^14^ The use of modeling and simulation approaches, including physiologically‐based pharmacokinetic (PBPK) models, provide a unique opportunity for closing these knowledge gaps through integrating available *in vitro* and clinical information to manage complex DDIs associated with LNG and to enhance decision making.^15^ PBPK models are routinely used in drug development and regulatory evaluation to evaluate the impact of DDIs on the exposure of victim drugs under clinically relevant conditions.

The Center for Pharmacometrics and Systems Pharmacology at the University of Florida and the Bill & Melinda Gates Foundation have agreed to collaborate on studying the impact of DDIs on the efficacy and safety of hormonal contraceptives.^15^ The proposed research strategy rests on the integration of exposure data from PBPK‐based DDI models, dose‐response relationships derived from model‐based meta‐analysis, and real‐world outcomes data utilizing interdisciplinary and interinstitutional collaboration among academia, industry, and nonprofit organizations. This paper is the first in a series of papers that focuses on the development and verification of PBPK models that study the impact of DDIs on exposure and PKs of hormonal contraceptives.

The aim of this study was to develop a PBPK framework to evaluate DDIs of oral hormonal contraceptives using LNG as the victim drug. Focusing on CYP3A4 as the major interacting pathway for CHCs, we developed PBPK models for LNG and its common comedication EE using previous DDI studies.^16^, ^17^, ^18^ The PBPK platform for CHCs was then applied to investigate the impact of DDIs on the PKs of LNG in different clinically relevant scenarios.

## METHODS

### Software and parameter estimation algorithm

PBPK modeling was performed in PK‐Sim and MoBi version 8.0. Parameter optimization was accomplished using the built‐in Monte‐Carlo algorithm. Software and data are available on Github (https://github.com/Open‐Systems‐Pharmacology), an open source platform for joint development, review, qualification, and application of state‐of‐the‐art tools for PBPK and Systems Pharmacology modeling and an open library of models and data.

### Model development and verification

**Figure 1:**
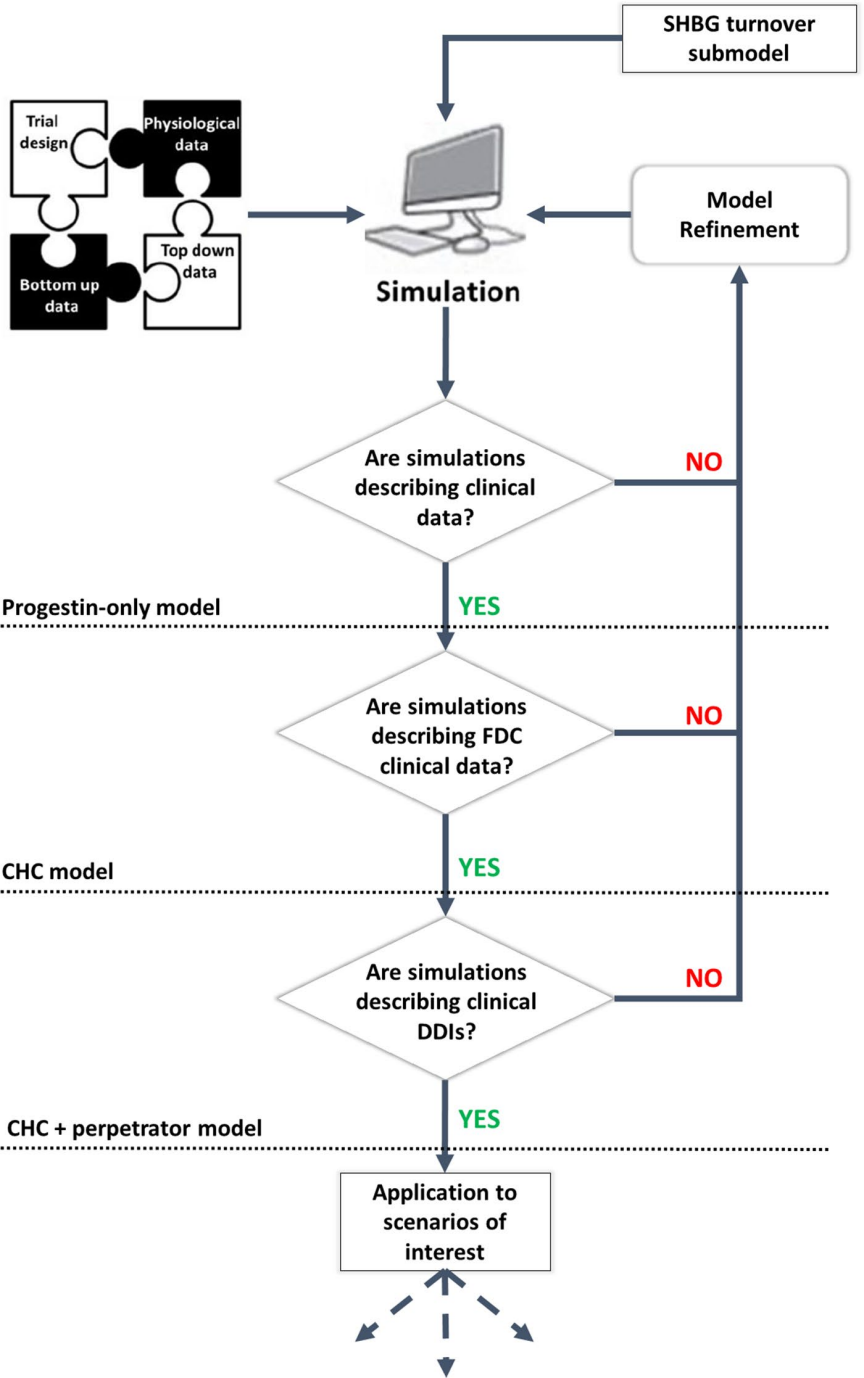
Overview of our PBPK modeling strategy. The simulated LNG fraction metabolized by CYP3A4 (fm_CYP3A4_) was verified by comparing to published clinical drug‐drug interaction (DDI) studies.^16^, ^17^, ^18^ CHC, combined hormonal contraceptive; FDC, fixed‐dose combination; PBPK, physiologically‐based pharmacokinetic; SHBG, sex hormone binding globulin.

Model development and verification was performed using a step‐wise approach (**Figure** **1**). The simulated LNG fraction metabolized by CYP3A4 (fm_CYP3A4_) was verified by comparing to published clinical DDI studies.^16^, ^17^, ^18^ All model simulations, unless otherwise noted, utilized a virtual population of 20 women and mean PK metric values for simulation and verification purposes.

### Development and verification of a PBPK model for levonorgestrel‐only contraceptive in normal BMI women

The LNG PBPK model was developed using the PK‐Sim population database to inform the dependence of anatomic and physiological parameters, such as organ volumes, blood flow rates, surface permeabilities or tissue composition based on age, sex, and BMI. Relative tissue distribution of CYP3A4 was implemented into the model from the PK‐Sim expression reverse transcription‐polymerase chain reaction database derived from Nishimura *et al*.^19^ Systemic levels of albumin and SHBG were based on the built‐in expressed sequence tags database and on the clinical results by Kuhnz *et al*.^13^

Drug‐specific parameters characterizing LNG ADME properties were identified by combining *in vitro* and clinical data. LNG molecular weight and logP were obtained from the literature. Unbound fraction (*f*_u_) was set as a maximum effect (E_max_) function of LNG binding kinetics to systemic albumin and SHBG. Tissue‐to‐plasma partitioning coefficients were calculated using the model developed by Rodgers and Rowland.^20^, ^21^ The base disposition model developed with data after 0.09 mg i.v. LNG administration was further expanded by incorporating aqueous solubility, intestinal permeability, and drug dissolution to simulate LNG exposure after oral administration. LogP (as lipophilicity), specific intestinal permeability, and dissolution‐related parameters were then optimized by comparing model prediction with observed plasma PK data following oral administration of 0.09 mg of an LNG‐only formulation to women with BMI < 30 kg/m^2^.^22^ Due to the lack of *in vitro* phenotyping data and drug interaction with strong inhibitors on the PKs of LNG as stand‐alone contraceptive product, contribution of CYP3A and other metabolic pathways of LNG were parameterized using LNG‐EE data for telithromycin (see below).

LNG systemic exposure predicted by the PBPK model was externally verified with additional datasets not used during the model‐building process, after oral administration of higher (0.27 mg) and lower doses (0.03 mg) of LNG‐only formulations in normal BMI women^22^ (see **Supplementary Material** for study details). Model‐predicted mean along with 5th to 95th percentiles were compared with respective clinical observations. Further model verification and adequate goodness‐of‐fit was determined by ensuring ratios of model‐predicted to clinically observed PK metrics (e.g., area under the curve (AUC), volume of distribution at steady‐state (V_ss_)) were between 0.8 and 1.25.

### Parameterization of enzyme contribution of LNG metabolism

The contribution of CYP3A4 to unbound LNG hepatic clearance was back‐calculated from a clinical DDI study comparing the systemic exposure of a combined LNG‐EE contraceptive administered with and without the strong CYP3A4 inhibitor telithromycin (i.e., net LNG fm_CYP3A4_ when administered in combination with EE).^6^ However, LNG metabolic clearance was estimated to be ~ 30% lower for the CHC compared with LNG‐only formulations.^23^ Only a fraction of the LNG metabolic pathway (70%) was subject to a subsequent interaction with a second strong CYP3A4 inhibitor, namely telithromycin. Thus, the back calculated area under the concentration‐time curve ratio (AUCR) resulting from the concomitant interaction on LNG exposure (i.e., EE and telithromycin) would be 2.1 (i.e., AUCR/0.7), which is equivalent to an LNG fm_CYP3A4_ of 47%. In addition to unbound intrinsic clearance by CYP3A4, nonspecific hepatic clearance and protein binding kinetics for albumin and SHBG were estimated by fitting the model to subject‐level data following i.v. administration of 0.09 mg of LNG‐only contraceptive to 18 women.^22^ Given the lack of mechanistic information, we lumped the contribution of phase II enzymes to LNG and EE clearance into a nonspecific hepatic clearance.

### Development and verification of a PBPK model for combined LNG‐EE contraceptives in normal BMI women

The system‐specific components of the verified PBPK model for LNG remained unchanged. EE was added as a new compound to simulate the combined progestin‐estrogen administration. The molecular weight, logP, aqueous solubility, and *f*_u_ of EE were obtained from literature. In contrast to LNG, EE does not bind to SHBG resulting in a constant *f*_u_. Tissue‐to‐plasma partitioning coefficients were calculated using the equation developed by Rodgers and Rowland.^20^, ^21^ Total hepatic clearance and specific organ permeability were estimated by fitting the model to subject‐level data following i.v. administration of 0.06 mg of ^13^C‐ethinyl estradiol formulation to 17 women.^22^ The base disposition model developed following i.v. dosing was further expanded to the oral administration by incorporating aqueous solubility, intestinal permeability, and drug dissolution. LogP (as lipophilicity), specific intestinal permeability, and dissolution‐related parameters were optimized by comparing model prediction with observed plasma PKs following oral administration of 0.02 mg of an EE formulation to normal BMI women.^22^

An E_max_ function was implemented to account for the observed net effect of EE on SHBG concentrations of ~ 2.3‐fold.^13^ EE concentration needed to elicit 50% of the maximum effect on SHBG was estimated using data from clinical trials after multiple administrations of the combined drug product.^13^ Note that LNG itself can affect SHBG levels, thus affecting the observed net effect on SHBG and warranting two different E_max_ values depending on EE alone or EE in combination with LNG.^23^ The internal turnover model for SHBG was completed by inputting a mean degradation terminal half‐life of ~ 7 days, estimated from the observed decrease in SHBG postpartum levels studied during the first 10 days after delivery.^24^ A competitive CYP3A4 inhibition was implemented into the EE PBPK model following the comprehensive analysis carried out by Reinecke *et al*.^23^ A dissociation constant for the inhibitor‐enzyme complex of 10 pmol/L was estimated to account for a 30% reduction in LNG fm_CYP3A4_.^24^ The performance of the combined PBPK model was evaluated by replicating the study design by Kuhnz *et al*.^13^ after single and multiple administrations (21 days) of a triphasic oral contraceptive (i.e., ascending doses of LNG combined with EE) and comparing the predicted and observed PK profiles (**Figures** **S11 and S12**).

The lack of PK data following administration of EE as monotherapy precluded the direct external verification of this PBPK model. Thus, clinically observed plasma concentration profiles of LNG and EE following single as well as multiple oral administrations of the CHC were compared with model‐predicted outputs.^13^ Adequate goodness‐of‐fit was concluded when the ratios between predicted and observed AUC values were between 0.8 and 1.25.

An additional model verification step was performed comparing predicted and observed AUC values for LNG, when administered as a combined contraceptive, with and without three different strong CYP3A4 inducers (carbamazepine, rifampicin, and efavirenz). An E_max_ function was implemented to the rifampicin PBPK model, to account for the observed stimulatory effect of rifampicin on SHBG concentrations of approximately twofold.^18^ Available verified perpetrator models of efavirenz (https://github.com/Open‐Systems‐Pharmacology/Efavirenz‐Model/releases) and rifampin (https://github.com/Open‐Systems‐Pharmacology/Rifampicin‐Model/releases) were used to predict DDIs. For carbamazepine, a PBPK model was developed during this project (details about carbamazepine PBPK model development and verification can be found in the **Supplementary Material**). The PBPK model was considered externally verified when the ratios between the predicted and observed LNG AUC were contained within 0.8 and 1.25.

### Local sensitivity analysis

Sensitivity of the final drug‐specific LNG model parameters to AUC from zero to infinity (AUC_0–∞_) was evaluated if they were optimized or showed high uncertainty when obtained experimentally. Sensitivity of a parameter was calculated as a ratio of AUC change to the relative variation of that parameter, as previously reported by Hanke *et al*.^25^ Briefly, local parameter sensitivity analysis was conducted by varying each parameter of interest by +/− 10%, while keeping all other parameters constant, and recording the respective AUC_0–∞_ change. Statistically, this leads to a sensitivity unit of 1 to be interpreted as for each 10% increase in the specified parameter, a 10% increase in AUC_0–∞_ is expected. Results of the sensitivity analysis can be found in the **Supplementary Material** **Figure** **S10**.

### DDI simulations

After verifying the adequacy of the back‐calculated LNG fm_CYP3A4_ against three different strong inducers, the model was applied prospectively to predict the impact of strong CYP3A4 inhibitors on LNG systemic exposure. DDI simulations were conducted for clarithromycin and itraconazole, two strong CYP3A4 inhibitors, considering their different mechanisms of interaction (i.e., mechanism‐based and competitive interactions). Verified PBPK models for the perpetrators were developed by Hanke *et al*. and are available on Open Systems Pharmacology (https://github.com/Open‐Systems‐Pharmacology/Clarithromycin‐Model and https://github.com/Open‐Systems‐Pharmacology/Itraconazole‐Model).^25^ Itraconazole simulations included 100 mg and 200 mg q.d. dosing scenarios, whereas clarithromycin simulations utilized 250 mg and 500 mg b.i.d., mimicking the most common therapeutic regimens for both perpetrators.

### Application of the LNG‐only and combined LNG‐EE PBPK models to predict the impact of DDI in obese women

The developed LNG‐only and combined LNG‐EE PBPK models were used to simulate LNG concentration‐time profiles in obese women (i.e., BMI ≥ 30 kg/m^2^). Algorithms incorporated in PK‐Sim allow for the adjustment of various system parameters with respect to absorption, distribution, metabolism, and excretion processes based on BMI, including increased fat, skin, muscle volumes, organ blood flow, cardiac output, and organ volume. To avoid unrealistic parameter generation and ensure sources of physiological and biochemical interindividual variability in system parameters are properly incorporated into the model during this process, the randomly assigned body weight based on BMI dictates the position of the individual on the probability distribution of subsequent correlated parameters (e.g., organ volume).^26^ Two different studies available in the literature were replicated^14^, ^27^ as a form of additional external verification. The model was deemed appropriate when the ratios between predicted and observed AUC values were between 0.8 and 1.25.

The PBPK models were also applied in an exploratory study to prospectively simulate the impact of obesity‐related decreases in SHBG levels for the DDIs presented in this work for normal BMI (< 25 kg/m^2^) women (i.e., LNG‐EE combined and LNG‐only contraceptives at steady‐state conditions) and considering multiple therapeutic doses of the selected perpetrators (rifampicin, carbamazepine, efavirenz, and itraconazole). Last, various LNG PK metrics were statistically compared for normal BMI and obese women populations.

## RESULTS

### Model development and verification

**Figure 2:**
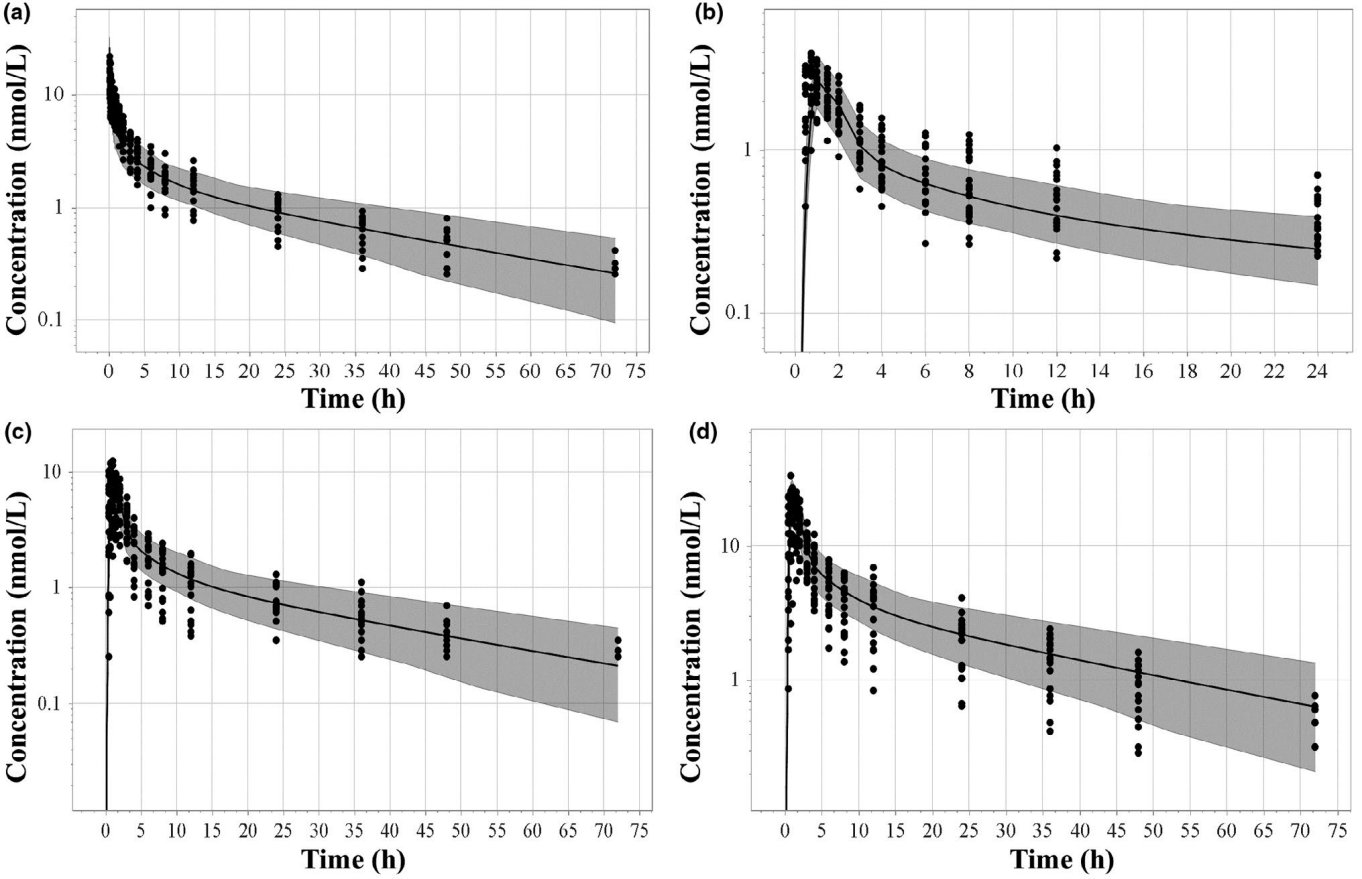
Simulated and observed (solid circles^22^) concentration‐time curves following intravascular bolus administration of 0.09 mg (a) and oral administration of 0.03 mg (b), 0.09 mg (c), and 0.27 mg (d) of LNG‐only formulations. Shaded area represents the predicted 5th to 95th percentile range. Note the oral 0.03 mg (b) and 0.27 mg (d) data were not used for model optimization and are thus considered external validation. LNG, levonorgestrel.

The final PBPK model parameters for LNG are summarized in **Table** **1**. Local sensitivity analysis indicated that fraction unbound is a key model parameter with a sensitivity value of −1.01. No other parameters showed a sensitivity above an absolute value of 1. A complete summary of the sensitivity analysis results is provided in **Figure** **S10**. After i.v. administration, the *V*_ss_ for LNG was estimated to be ~ 1.7 L/kg, which was comparable to the observed range of 1.4–2.1 L/kg in adult women.^13^, ^28^ However, predicted interindividual clearance variability was smaller than the one estimated via noncompartmental analysis from the observed dataset (i.e., 35% vs. 48%).^22^ Convergence between predicted and observed subject‐level PK profiles after i.v. and oral administration of different doses of LNG are shown in **Figure** **2**. Observed data were contained within the simulated 5th to 95th percentile range, demonstrating the validity of the full PBPK model to capture LNG plasma concentrations when drug is administered as monotherapy.

**Table 1:** Final LNG PBPK model parameters

LNG drug‐specific input parameters		
Parameter	Value	Source
Molecular weight	312.4 g/mol	^39^
Lipophilicity	4.00	Fitted
Protein binding: albumin	K_off_: 6.50/minute K_d_: 6 µmol/L	Fitted
Protein binding: SHBG	K_off_: 17.4/minute K_d_: 0.0005 µmol/L	^33^
Aqueous solubility	2.05 mg/L	^40^
FaSSIF solubility	14.0 mg/L at 6.5 pH	^40^
Cl_specific_/CYP3A4	1.45 µL/minute/pmol CYP3A4	Fitted
Other hepatic clearance	0.10 L/h/kg	Fitted
Specific intestinal permeability	0.0002 cm/minute	Fitted
Specific organ permeability	0.31 cm/minute	Fitted
Partition coefficients	Rodgers and Rowland	
Dissolution		
LNG 0.03, 0.09, 0.15, 0.27 mg T_80%_	30 minutes	^40^
LNG 0.75 mg T_80%_	75 minutes	Fitted
LNG 1.50 mg T_80%_	180 minutes	Fitted
LNG 0.03, 0.09, 0.15, 0.27, 0.75, 1.50 mg Lag time	10 minutes (0.75, 1.50 = 0 minutes)	Fitted

Note

that due to the fitting of albumin K_d_, there may exist identifiability issues in the fitted K_off_ and K_on_ values. Specific intestinal permeability refers to the compound’s transcellular permeability of the intestinal wall. Specific organ permeability refers to the compound‐dependent lipid bilayer permeability.

FaSSIF, fasted state simulated intestinal fluid; K_d_, dissociation constant; K_off_, rate of protein‐ligand dissociation; K_on_, rate of protein‐ligand binding; LNG, levonorgestrel; PBPK, physiologically‐based pharmacokinetic; SHBG, sex‐hormone binding globulin.

The final model parameters for EE used in the combined LNG‐EE PBPK model are summarized in **Table** **2**. Simulated vs. observed AUCR fell within the acceptance range (**Table** **S5**), except for EE AUC after 21‐day treatment cycle (i.e., AUC predicted/AUC observed = 1.38).^13^ The adequacy of the model to capture the DDI between LNG and EE when administered together as a single dose or multiple dose formulations was further confirmed via predicted plasma concentration vs. time plots overlaid with observed data (**Figures** **S11** **–S13**). **Table** **3** summarizes the AUCRs for LNG when the combined LNG‐EE formulation is administered with and without three CYP3A4 inducers carbamazepine, rifampicin, and efavirenz. Because the AUCRs were rather close to clinical observations the combined LNG‐EE PBPK model was considered adequate for its purpose and applied to prospective simulations.

**Table 2:** Final EE PBPK model parameters

EE drug‐specific input parameters		
Parameter	Value	Source
Molecular weight	296.4 g/mol	^32^
Lipophilicity	3.6	Fitted
Protein binding: nonspecific	1.5%	^32^
Aqueous solubility	3.9 µg/mL	^41^
Total hepatic clearance	4.1 mL/minute/kg	Fitted
Specific intestinal permeability	1.0 × 10^−4^ cm/s	Fitted
Specific organ permeability	0.001 cm/minute	Fitted
Partition coefficients	Rodgers and Rowland	
CYP3A4 inhibition		
K_i_	2 pmol/L	^24^
Net SHBG induction (LNG:EE)		
EC_50_	1 pmol/L	Fitted
E_max_	2.3	Fitted
SHBG induction (EE)		
EC_50_	1 pmol/L	Fitted
E_max_	2.25	Fitted
Dissolution		
T_80%_	40 minutes	Fitted
Lag time	10 minutes	Fitted

Specific intestinal permeability refers to the compound’s transcellular permeability of the intestinal wall. Specific organ permeability refers to the compound‐dependent lipid bilayer permeability.

EC_50_, 50% of the maximum effect; EE, ethinyl estradiol; E_max_, maximum effect; K_i_, inhibition constant; LNG, levonorgestrel; PBPK, physiologically‐based pharmacokinetic; SHBG, sex‐hormone binding globulin.

**Table 3:** Summary of LNG AUCR (predicted vs. observed ratios for AUC_0–t_) for the following DDI scenarios: CBZ (600 mg q.d.) and LNG (0.250 mg) + EE (0.05 mg)^16^; EFA (600 mg q.d.) and LNG (0.75 mg single dose)^17^; RFP (600 mg q.d.), and LNG (0.03 mg)^18^

Formulation	Observed AUCR	Predicted AUCR	Predicted/observed
CBZ (600 mg QD)/LNG + EE	0.65	0.66	1.01
EFA (600 mg QD)/LNG	0.42	0.46	1.09
RFP (600 mg QD)/LNG	0.43	0.42	1.02

AUC_0–t_, area under the plasma concentration‐time curve from time point 0 to the end of the dosing interval; AUCR, area under the concentration‐time curve ratio; CBZ, carbamazepine; DDI, drug‐drug interaction; EE, ethinyl estradiol; EFA, efavirenz; LNG, levonorgestrel; RFP, rifampin.

### Model application to clinical scenarios with CYP3A4 inhibition and in obese women

Model simulations investigating the impact of CYP3A4 inhibitors on systemic exposure of LNG in women with normal BMI showed that itraconazole and clarithromycin slightly increased LNG exposure; AUCR between 1.2 and 1.4 for LNG‐only and between 1.2 and 1.5 for LNG‐EE formulations (see **Table** **S9** for detailed results).

**Figure 3:**
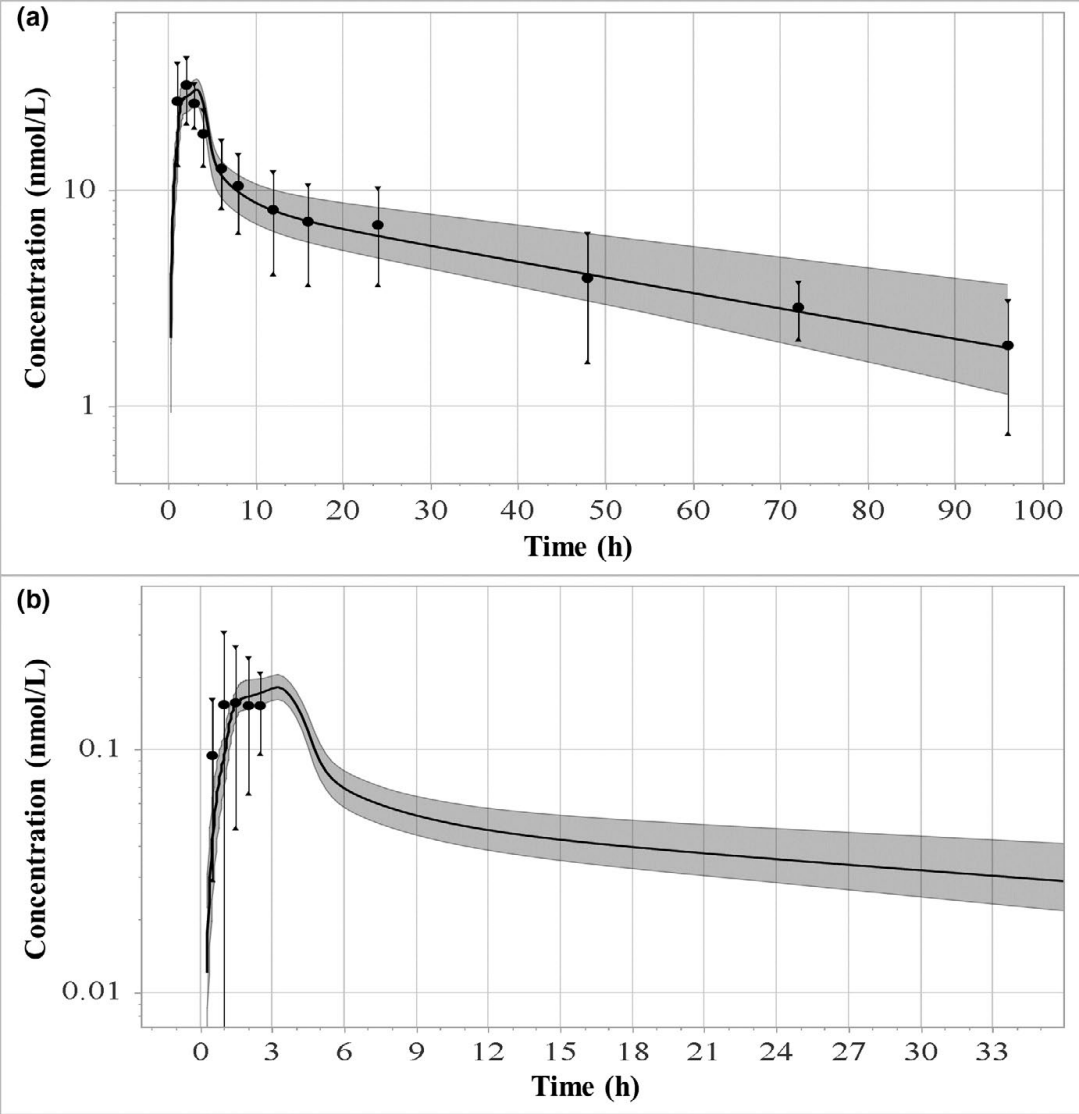
Simulated and observed (solid circles^14^, ^29^) total (a) and unbound (b) LNG concentration‐time curves following oral administration of 1.5 mg LNG‐only formulation in obese women. LNG, levonorgestrel.

**Figure** **3a** shows the predicted and observed LNG plasma profiles after the administration of 1.5 mg of LNG alone in obese women. The model was able to capture the impact of BMI on LNG exposure. Predicted mean apparent *V*_ss_ and median terminal half‐life in obese women were in line with their observed counterparts, 4.65 vs. 4.35 L/kg and 43 vs. 41 hours,^14^ which are significantly higher than the respective values observed in women with normal BMI. Simulated vs. observed total LNG AUCR was ~ 0.95 in obese women. The LNG‐only PBPK model captured the observed 1.7‐fold reduction in total LNG exposure in obese women^14^ as well as the mean unbound LNG C_max_ in normal BMI (0.10 vs. 0.09 ng/mL) and obese women (0.06 vs. 0.07 ng/mL), measured by Edelman *et al*.^29^ (**Figure** **3b**). Following oral administration of 150 µg LNG and 30 µg EE, simulated to observed total LNG AUCR was ~ 0.78 in obese women. However, when interindividual variability was considered, simulated and observed AUCRs overlapped (62 ± 32 ng*h/mL vs. 45–142 ng*h/mL).^14^, ^27^, ^29^ Moreover, after 21‐day treatment cycle, simulated AUC for total LNG in normal BMI and obese women showed similar SHBG levels (70.1 vs. 66.6 nmol/L, *P* = 0.75), which were not statistically different, in line with the clinical report.^27^ On the other hand, in an obesity‐related decrease in SHBG concentrations (27.6 vs. 55.4 nmol/L, *P* = 0.037), total LNG AUC would decrease by ~ 1.7‐fold in obese women (*P* value < 0.01).^14^

**Figure 4:**
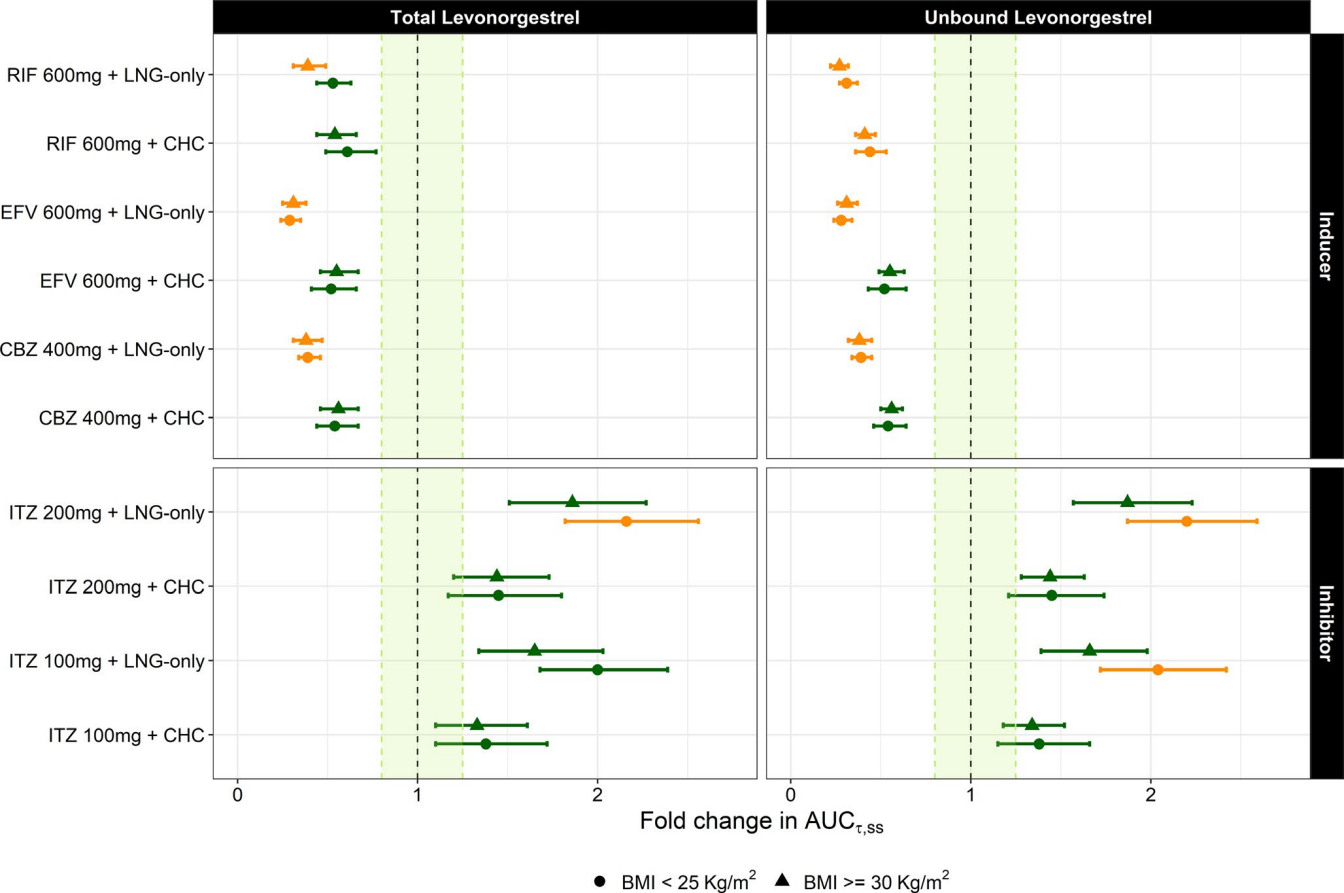
Simulated LNG fold change in AUC (mean with 95% CIs) following oral administration of 150 µg LNG‐only contraceptive or in combination with 30 µg EE (CHC) to women showing BMI < 25 kg/m^2^ and obese women (BMI ≥ 30 kg/m^2^) who have also been treated with strong CYP3A4 perpetrators. Green points indicate a < 2‐fold change whereas yellow indicates a 2 to 5‐fold change. Results are expressed in terms of total and unbound LNG exposure. AUC_r,ss_, area under the concentration‐time curve ratio at steady‐state; BMI, body mass index; CBZ, carbamazepine; CHC, combined hormonal contraceptive; CI, confidence interval; EE, ethinyl estradiol; EFV, efavirenz; ITZ, itraconazole; LNG, levonorgestrel; RIF, rifampicin.

DDI simulations of either 150 µg LNG‐only or in combination with 30 µg EE in normal BMI women showed significant changes in exposure. For example, total LNG exposure decreased from 18.9 to 9.95 µmol*minute/L when CHC administration was concomitant with 400 mg of carbamazepine (CBZ). Comparatively, the LNG‐only formulation had total exposure decrease from 7.22 to 2.77 µmol*minute/L when administered with 400 mg of CBZ. Total LNG exposure increased from 18.9 to 25.6 µmol*minute/L when the CHC was combined with 100 mg of itraconazole (ITZ), similarly, exposure increased from 7.22 to 14.6 µmol*minute/L when the LNG‐only formulation was combined with 100 mg of ITZ. Unbound LNG exposure showed similar magnitudes of change as the total exposure. The same LNG dosing and DDI scenarios in obese women also showed significant changes in total exposure. LNG exposure decreased from 9.06 to 4.98 µmol*minute/L with concomitant CHC and CBZ administration, whereas exposure decreased from 5.05 to 1.78 µmol*minute/L when the LNG‐only formulation was administered with 400 mg of CBZ. For inhibitor DDI scenarios in obese women, exposure increased from 9.06 to 12.0 µmol*minute/L after concomitant CHC administration with 100 mg of ITZ, whereas exposure increased from 5.05 to 7.78 µmol*minute/L when the LNG‐only formulation was combined with 100 mg of ITZ. Unbound LNG exposure showed similar relative magnitudes of change when compared with the normal BMI women population. **Figure** **4** shows the fold AUC change comparing the normal BMI LNG AUC reference (no DDI) to the AUC after various DDI scenarios in normal BMI women as well as the same for obese women (i.e., using the obese BMI AUC reference for fold AUC change in obese women). Overall, these results show that although the magnitude in LNG exposure is different between normal BMI and obese women, the relative exposure change within DDI scenarios is very similar.

## DISCUSSION

This work reports the first integration of data from various clinical studies to develop a PBPK framework to study DDI scenarios for oral administrations of LNG‐alone and, more importantly, combined LNG with EE (**Figures** **1** and **2**). This is an important achievement for better understanding the safe use of hormonal contraceptive products. As there was not enough information on the fraction metabolized when LNG was administered as a single agent, the fraction metabolized was derived from a clinical DDI study comparing the systemic exposure of a combined LNG‐EE contraceptive administered with and without telithromycin.^30^ The resulting 1.5‐fold increase in the extent of exposure observed for the LNG component in the CHC when co‐administered with telithromycin suggests a contribution of CYP3A4 to LNG hepatic clearance of ~ 33%.^6^ EE was reported to also inhibit CYP3A4, suggesting that LNG clearance is ~ 30% lower following coadministration of EE compared to LNG alone.^24^ LNG fm_CYP3A4_ back‐calculated from the reported clinical DDI with telithromycin was consequently corrected for the confounding effect of EE on this metabolic pathway, resulting in a net fm_CYP3A4_ of 47% for LNG‐only contraceptives. In general, the model is able to adequately describe observed AUCR for both LNG and EE based on predefined criteria of 0.8–1.25‐fold, except for EE AUC after 21‐day treatment cycle (i.e., AUC simulated/AUC observed = 1.38). This mismatch is likely due to the high uncertainty around the observed AUC (864 ± 739 pg*h/mL), which can be further explained by the poor sensitivity of the bioanalytical method used by the authors to quantify serum EE levels (e.g., low limit of quantification was equivalent to 20% of the measured C_max_). The simulated AUC for EE during the last dosing interval of the 21‐day treatment cycle (1,198 pg*hour/mL) fell within the 95% confidence interval back‐calculated for the observed AUC mean.

When administered in combination with EE, LNG fm_CYP3A4_ is ~ 33%, which is significantly lower than that of other progestins, such as norgestimate and drospirenone, whose fm_CYP3A4_ approximates 60%.^6^, ^31^, ^32^ Therefore, LNG is less likely to be impacted by CYP3A4 DDIs relative to contraceptive drugs with higher fm_CYP3A4_, particularly when co‐administered with strong CYP3A4 perpetrators. It should be noted that the current EE elimination model was implemented as total hepatic clearance, with no explicit CYP3A4‐mediated clearance. Therefore, CYP3A4 perpetrators do not influence LNG exposure via EE in our model. We do not expect this indirect interaction to be significant given that currently available clinical DDI data were characterized reasonably well by our model via the direct interaction with LNG’s metabolism. On the other hand, LNG, but not norgestimate or drospirenone, shows high affinity to binding to SHBG in plasma.^33^, ^34^ Plasma SHBG concentrations can be influenced by drugs, such as LNG, EE, and rifampin, and are thus subject to dynamic changes, which can affect unbound LNG concentrations and LNG clearance. With this additional mechanism in mind, LNG’s risk of being impacted by DDIs may in fact be higher than expected based on CYP3A4‐mediated DDIs only. Simulated co‐administrations of LNG and clarithromycin or itraconazole (i.e., strong CYP3A4 inhibitors), resulted in a relatively weak interaction (≥ 1.25‐fold and < 2‐fold) according to current regulatory recommendations, whereas the CYP3A4 inducer DDI results indicated a moderate interaction with strong CYP3A4 inducers (≥ 50% and < 80%), according to regulatory recommendations.^35^ Based on PKs only, our results support the class labeling recommendations issued by the FDA, at least for LNG‐like drugs, in that CYP3A4 inducers cause a higher impact on systemic exposure of the victim drug.^35^

An important role of labeling recommendations is to identify optimal dosing regimens for subgroups of patients. To this end, we explored the potential impact of metabolic DDIs in obese women taking LNG alone or in combination with EE. Simulated changes in relative exposure due to strong CYP3A4 inducer or inhibitor administration were similar in normal BMI and obese women (**Figure** **4**). However, the impact on absolute LNG exposure was significantly different between both groups. In normal weight women, mean total LNG AUC was 18.9 and 7.22 µmol*minute/L for CHC and LNG‐only formulations, respectively. In comparison, obese women had mean total LNG AUCs of 9.06 and 5.05 µmol*minute/L for CHC and LNG‐only formulations, respectively. Unbound LNG AUCs were also different between normal weight (0.048 µmol*minute/L) and obese women (0.034 µmol*minute/L) after CHC administration. This result is expected because obesity seems to affect the hepatic clearance of LNG by increasing the fraction unbound, via reduced SHBG levels,^27^, ^29^ and not by affecting unbound clearance.^14^ In comparison, total and unbound LNG AUC in normal BMI women decreased to 9.95 and 0.025 µmol*minute/L when 400 mg CBZ is concomitantly administered with CHC, whereas total and unbound LNG AUC increased to 25.6 and 0.065 µmol*minute/L when 100 mg ITZ is concomitantly administered with CHC. This equates to an approximate 0.4‐fold decrease and 1.4‐fold increase in both total and unbound LNG AUC in normal BMI women within strong inducer and inhibitor DDIs, respectively. When evaluating DDIs in obese women, total and unbound LNG AUC decreased to 4.98 and 0.019 µmol*minute/L when 400 mg of CBZ is concomitantly administered with CHC, whereas total and unbound LNG AUC increased to 12.0 and 0.046 when 100 mg ITZ is concomitantly administered with CHC. These changes equate to an approximate 0.4‐fold decrease and 1.4‐fold increase in total and unbound LNG AUC in obese women within strong inducer and inhibitor DDIs, respectively. From this evidence we can conclude that obese women have decreased LNG exposure when compared with normal BMI women exposure, mostly likely due to higher *V*_ss_ observed in obese women resulting from significant compound distribution to adipose tissue. This larger *V*_ss_ explains the prolonged half‐life in obese women as well. However, as shown in **Figure** **4**, whereas absolute exposure may be different between normal BMI and obese women, the relative impact of DDIs between both groups is generally equivalent.

Whether the more pronounced magnitude of exposure change in obese women compared with normal BMI women translates into increased risks of unintended pregnancy in obese women is still subject of debate. If only free, unbound LNG contributes to the pharmacological action, the relative risks of unintended pregnancy due to DDI would be similar in obese and normal weight women, however, absolute risk may differ. On the other hand, if the “bioavailable hormone hypothesis” (i.e., free + albumin‐LNG concentrations, due to the low affinity binding, the progestin can dissociate from albumin in the tissue capillaries and effectively be available for biological activity) or the megalin‐mediated internalization of SHBG‐LNG complex into target cells, obese women would be under higher risks of contraceptive failure.^14^, ^36^, ^37^, ^38^ Both free and bioavailable hormone hypothesis will be tested within a complementary research project, focusing on exposure‐response relationship for LNG in terms of unintended pregnancy via model‐based meta‐analysis.

In conclusion, different intrinsic and extrinsic factors may influence systemic drug exposure of hormonal contraceptives. Addressing all potential permutations clinically is neither time nor cost‐effective. Leveraging PBPK models and prior clinical knowledge may provide a useful alternative to explore untested clinical scenarios. The strategic development of projects aimed at integrating methodologies from different disciplines may bridge preclinical and clinical drug development as well as build a bridge between academic curiosity and clinical practice. Under this collaborative philosophy, additional research on the clinical relevance of the simulated changes in the systemic exposure of LNG are being conducted using model‐based meta‐analysis and real‐world pharmacoepidemiologic analyses by different stakeholders involved in this multidisciplinary collaborative effort^15^ for the objective of evaluating the impact of DDI on the efficacy and safety of hormonal contraceptive agents. This combined approach allows for the identification of minimum effective exposure thresholds, which can be further applied as a target for the development of new contraceptive formulations and different routes of administrations, such as long‐term injectables.

## Funding

This project was funded by a grant (OPP1185454) provided by the Bill & Melinda Gates Foundation.

## Conflict of Interest

T.W., J.H., and H.W. are employees of BAYER AG. All other authors declared no competing interests for this work.

## Author Contributions

S.S., A.C., V.V., K.L., B.C., R.C., J.H., T.W., and H.W. wrote the manuscript. R.C. and S.S. designed the research. R.C., B.C., and K.L. performed the research. R.C., B.C., and K.L. analyzed the data.

## Supporting information

Supplementary Material
