# Supplementary material for: Quantitative Assessment of Levonorgestrel Binding Partner Interplay and Drug‐Drug Interactions Using Physiologically Based Pharmacokinetic Modeling
Source: CPT Pharmacometrics Syst Pharmacol. 2020 Dec 13;10(1):48–58. doi: 10.1002/psp4.12572 (PMC7825189; doi:10.1002/psp4.12572)
Supplement: Supplementary file 1 — Supplementary Material [file PSP4-10-48-s001.docx]

**Quantitative assessment of levonorgestrel binding partner interplay and drug-drug interactions using physiologically based pharmacokinetic modeling**

Supplemental Information

**Section 1: Carbamazepine PBPK Model Development and Qualification Tables and Figures**

Summary:

In order to verify the levonorgestrel model’s DDI simulation capabilities, a carbamazepine PBPK model was developed. The model was parameterized using a combination of literature-reported values and parameter estimation techniques using subject-level clinical data after intravascular and oral administration of carbamazepine obtained from Gérardin et al and Zhang et al. Optimization of the estimated parameters, when required, was determined using Monte Carlo simulations. The final drug-specific parameters for the verified carbamazepine model are summarized in Table S1.

The PBPK model verification was conducted by comparing predicted versus observed systemic concentration-time profiles obtained after administration of 200 and 400 mg of carbamazepine formulated as immediate release tablets as reported by Kohlmann et al. The dataset used to qualify the PBPK model had not guided the respective model development in order to show the external validity of the physiologically-based modeling approach.

In turn, the DDI model for carbamazepine was developed using an iterative bottom-up approach from mechanistic *in vitro* results reported by Vermet et al. The induction capability was qualified against clinical data showing the impact of 10 days of 800 mg QD of carbamazepine on midazolam exposure as reported by Backman et al. Predicted and observed AUCR values for midazolam in patients taking carbamazepine were 0.13 and 0.10, respectively. The midazolam PBPK model utilized for this verification analysis was developed in PK-Sim and was previously presented by Hanke et al.

Tables:

**TableS1.** Final Carbamazepine PBPK Model Input Parameters

**Table S2.** Summary of clinical trials used for parameter fitting and model qualification. PO = oral dosing, IV = intravenous dosing, SD = single dose, MD = multiple dose.

Table S3. Summary of predicted vs observed (Gérardin et al ) PK parameters for the 10 mg CBZ IV simulations.

Figures:

Figure S1. CBZ concentration vs time plot for the simulated oral administration of 200 mg of CBZ overlaid with the clinical trial data reported by Kohlmann et al.

**Figure S2.** CBZ concentration vs time plot for the simulated oral administration of 400 mg of CBZ overlaid with the clinical trial data reported by Kohlmann et al.

| Drug-Specific Parameters | | | |
| --- | --- | --- | --- |
| Parameter | **Value** | **Source** |  |
| Molecular Weight | 236.3 g/mol | Zhang et al 2011 |  |
| Lipophilicity | 2.13 | Fitted |  |
| Fu | 24 % | FDA-Approved Label |  |
| Aqueous Solubility | 0.12 mg/L | Zhang et al 2011 |  |
| In vitro Vmax/CYP3A4 | 2.12 pmol/min/pmol CYP3A4 | Fitted |  |
| Specific Intestinal Permeability | 4.3x10^-4^ cm/s | Fitted |  |
| Specific Organ Permeability | 0.02 cm/min | PK-Sim Standard Method |  |
| Partition Coefficients | PK-Sim Standard Method |  |  |
| CYP3A4 Induction | | | |
| EC50 | 59 µmol/L | Vermet et al 2016 |  |
| Emax | 16 | Vermet et al 2016 |  |
| Dissolution | | | |
| Suspension mean particle radius | 10 µm | Zhang et al 2011 |  |
| IR tablet mean particle radius | 75 µm | Zhang et al 2011 |  |
| Type of particle size distribution | monodispersed |  |  |
| Thickness (unstirred water layer) | 30 µm |  |  |

Table S1. Final Carbamazepine PBPK Model Input Parameters. Specific Intestinal Permeability refers to the compound’s transcellular permeability of the intestinal wall. Specific Organ permeability refers to the compound-dependent lipid bi-layer permeability.

| Study | Number of Patients | Average Age [years] | Study Type | Carbamazepine Dose (Route) [mg] |
| --- | --- | --- | --- | --- |
| Gérardin et al | 2 | 43 - 49 | Absolute bioavailability | 10 mg SD (IV) |
| Kohlmann et al | 76 | Not reported | Meta-analysis | 200 mg SD (PO) |
| Kohlmann et al | 94 | Not reported | Meta-analysis | 400 mg SD (PO) |
| Crawford et al | 4 | 16 - 37 | DDI | 300 – 600 mg QD (PO) |
| Backman et al | 5 | 25 – 63 | DDI | 700 – 900 mg QD (PO) |

Table S2. Summary of clinical trials used for parameter fitting and model qualification. PO = oral dosing, IV = intravenous dosing, SD = single dose, MD = multiple dose.

| Pharmacokinetic Parameter | Mean Predicted | Mean Observed | Ratio (Pred/Obs) |
| --- | --- | --- | --- |
| CL/ F (L/h/kg) | 0.013 | 0.015 | **0.87** |
| Vss/F (L/kg) | 1.14 | 0.96 | **1.19** |

Table S3. Summary of predicted vs observed (Gérardin et al ) PK parameters for the 10 mg CBZ IV simulations.

Figure S1. CBZ concentration vs time plot for the simulated oral administration of 200 mg of CBZ overlaid with the clinical trial data reported by Kohlmann et al.

Figure S2. CBZ concentration vs time plot for the simulated oral administration of 400 mg of CBZ overlaid with the clinical trial data reported by Kohlmann et al.

**Section 2: Ethinyl Estradiol PBPK Model Development and Qualification Tables and Figures**

Tables:

**Table S4.** Summary of clinical trials used for parameter fitting and model qualification.

Table S5. Summary of predicted vs observed (provided by Bayer AG) AUC parameters for the oral and IV EE simulations.

Figures:

**Figure S3.** EE Concentration vs time plot for the simulated IV administration of 0.06 mg of EE overlaid with the clinical trial data from Bayer AG’s Hormonal contraceptive agents database (HCAD).

**Figure S4.** EE concentration vs time plot for the simulated oral administration of 0.02 mg BID of EE overlaid with the clinical trial data from Bayer AG’s Hormonal contraceptive agents database (HCAD).

**Figure S5.** SHBG concentration vs time plot for the simulated oral administration of 0.03 mg BID of EE alone overlaid with the clinical trial data from Limpongsanurak et al 1981.

| Study | Number of Patients | Average Age [years] | Study Type | Ethinyl Estradiol Dose (Route) [mg] |
| --- | --- | --- | --- | --- |
| Bayer AG | 19 | 20 - 35 | Absolute bioavailability | 0.06 mg MD (IV) and 0.02 mg MD (PO) |
| Limpongsanurak et al 1981 | 17 | 18 – 42 | PK and SHBG levels | 0.03 mg MD (PO) |

Table S4. Summary of clinical trials used for parameter fitting and model qualification.

| Pharmacokinetic Parameter | Ratio (Pred/Obs) |
| --- | --- |
| IV | |
| AUC­­_0-tau_ | 1.02 |
| AUC_0-inf_ | 0.99 |
| Oral | |
| AUC­­_0-tau_ | 0.86 |
| AUC_0-inf_ | 0.84 |

Table S5. Summary of predicted vs observed (provided by Bayer AG) AUC parameters for the oral and IV EE simulations.

Figure S3. EE concentration vs time plot for the simulated IV administration of 0.06 mg of EE overlaid with the clinical trial data from Bayer AG’s Hormonal contraceptive agents database (HCAD).

Figure S4. EE concentration vs time plot for the simulated oral administration of 0.02 mg BID of EE overlaid with the clinical trial data from Bayer AG’s Hormonal contraceptive agents database (HCAD).

Figure S5. SHBG concentration vs time plot for the simulated oral administration of 0.03 mg BID of EE alone overlaid with the clinical trial data from Limpongsanurak et al 1981.

**Section 3: Levonorgestrel PBPK Model Development and Qualification Tables and Figures**

Tables:

**Table S6.** Summary of clinical trials used for parameter fitting and model qualification. PO = oral dosing, IV = intravenous dosing, SD = single dose, MD = multiple dose, EE = Ethinyl Estradiol.

Table S7. Summary of predicted vs observed (Bayer AG HCAD) LNG PK parameters for the 0.09 mg IV levonorgestrel simulations.

Table S8. Summary of LNG Cmax and AUC_0-t_ predictions and observations (Bayer AG HCAD) based for each dose of the orally administered levonorgestrel simulations.

Figures:

Figure S6. LNG concentration vs time plot for the simulated IV administration of 0.09 mg of LNG overlaid with the clinical trial data (Bayer HCAD).

Figure S7. LNG concentration vs time plot for the simulated multiple dose oral administration of 0.03 mg of LNG overlaid with the clinical trial data (Bayer HCAD).

**Figure S8**. LNG concentration vs time plot for the simulated oral administration of a single dose of 0.15 mg of LNG overlaid with the external clinical data. Falcao et al group1 (blue), Falcao et al group2 (red), Kunhz et al (black).

**Figure S9.** LNG concentration vs time plot for the simulated oral administration of 0.75 mg of LNG overlaid with the external clinical data. Kook et al(black), He et al (red), Tremblay et al (blue).

**Figure S10**. Plot of the parameter sensitivity analysis with respect to AUC_0-∞_ for an oral administration of 0.03 mg QD of levonorgestrel.

| Study | Number of Patients | Average Age (SD) [years] | Study Type | Levonorgestrel Dose (Route) [mg] |
| --- | --- | --- | --- | --- |
| Bayer AG  HCAD | 21 | 41.2 (± 4.5) | Bioavailability | 0.03 (PO) SD & MD |
| Bayer AG HCAD | 12 | 25.8 (± 4.7) | Relative-Bioavailability | 0.15 (PO) SD (with 0.03 of EE) |
| Bayer AG HCAD | 18 | 32.2 (± 5.5) | Absolute-Bioavailability | 0.03 (PO) SD  0.09 (PO and IV) SD  0.27 (PO)SD |

Table S6. Summary of clinical trials used for parameter fitting and model qualification. PO = oral dosing, IV = intravenous dosing, SD = single dose, MD = multiple dose, EE = Ethinyl Estradiol.

| Pharmacokinetic Parameter | Predicted | Observed | Ratio (Pred/Obs) |
| --- | --- | --- | --- |
| AUC_0-∞_ (ng*h/mL) | 20.2 | 20.5 ± 8.2 | **0.99** |
| Half-Life (h) | 25.13 | 22.4 ± 7.6 | **1.12** |
| CL/ F (L/h/kg) | 0.07 | 0.06 ± 0.02 | **1.17** |
| Vss/F (L/kg) | 1.89 | 2.1 ± 1.2 | **0.90** |

Table S7. Summary of predicted vs observed (Bayer AG HCAD) LNG PK parameters for the 0.09 mg IV levonorgestrel simulations.

| Formulation | | C_max_ (ng/mL) | | AUC_0-t_ (ng*h/mL) | |
| --- | --- | --- | --- | --- | --- |
|  |  | **Mean ± SD** | **Ratio (Pred/mean-Obs)** | **Mean ± SD** | **Ratio (Pred/mean-Obs)** |
| LNG 0.03 mg | Predicted | 0.85 | **0.88** | 4.26 | **0.87** |
|  | Observed | 0.97 ± 0.41 |  | 4.91 ± 1.62 |  |
| LNG 0.09 mg | Predicted | 2.52 | **1.09** | 18.9 | **1.02** |
|  | Observed | 2.31 ± 0.86 |  | 18.46 ± 7.42 |  |
| LNG 0.27 mg | Predicted | 7.2 | **1.16** | 55.7 | **1.02** |
|  | Observed | 6.2 ± 1.93 |  | 54.5 ± 19.7 |  |

Table S8. Summary of LNG Cmax and AUC_0-t_ predictions and observations (Bayer AG HCAD) based for each dose of the orally administered levonorgestrel simulations.

Figure S6. LNG concentration vs time plot for the simulated IV administration of 0.09 mg of LNG overlaid with the clinical trial data (Bayer HCAD).

Figure S7. LNG concentration vs time plot for the simulated multiple dose oral administration of 0.03 mg of LNG overlaid with the clinical trial data (Bayer HCAD).

Figure S8. LNG concentration vs time plot for the simulated oral administration of a single dose of 0.15 mg of LNG overlaid with the external clinical data. Falcao et al group1 (blue), Falcao et al group2 (red), Kuhnz et al (black).

Figure S9. LNG concentration vs time plot for the simulated oral administration of 0.75 mg of LNG overlaid with the external clinical data. Kook et al(black), He et al (red), Tremblay et al (blue).


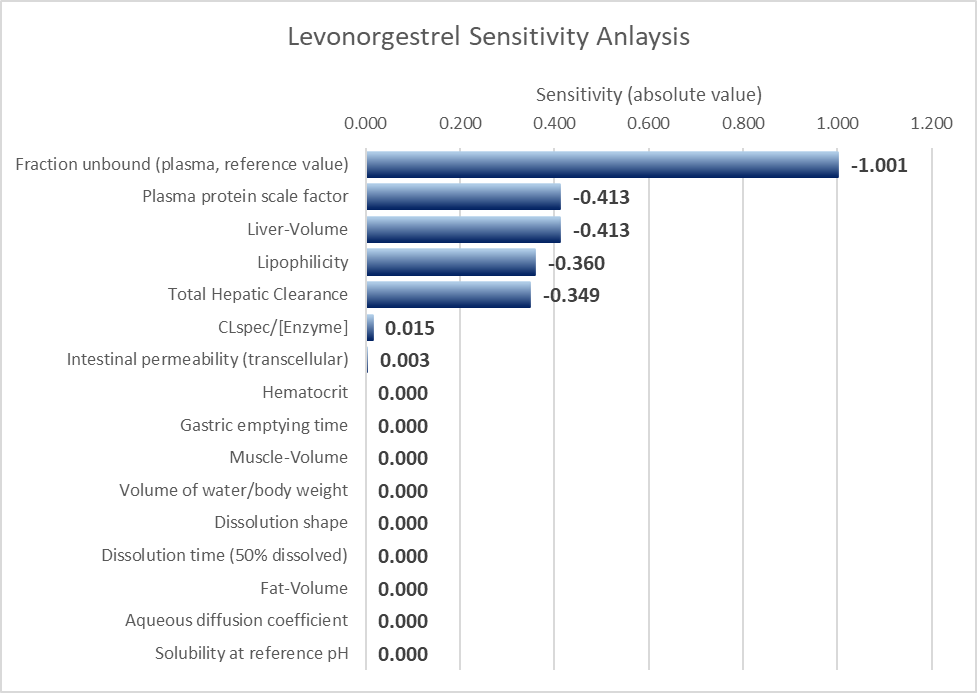


Figure S10. Plot of the parameter sensitivity analysis with respect to AUC_0-∞_ for an oral administration of 0.03 mg QD of levonorgestrel.

**Section 4: Levonorgestrel PBPK Model BMI and DDI Qualification Tables and Figures**

Tables:

**Table S9.** Summary of LNG AUCR (inhibited vs uninhibited ratios for AUC_0-t_ of PBPK model predictions for the itraconazole (ITRA) (100 or 200 mg QD) and LNG (0.03 mg) with and without EE (0.03 mg) DDI simulations as well as the clarithromycin (CLR) (250 or 500 mg QD) and LNG (0.03 mg) with and without EE (0.03 mg) DDI simulations.

**Table S10**. Summary of predicted vs observed LNG PK parameters, under fed and fasting conditions, for the 1.5 mg PO levonorgestrel simulations.

**Table S11**. Summary of predicted vs observed LNG clearance in normal BMI and obese patients for the 1.5 mg PO levonorgestrel simulations.

Figures:

Figure S11. Predicted and observed (Kuhnz et al) LNG concentration vs time plot after a single oral dose of 0.125 mg of levonorgestrel + 0.03 mg of ethinyl estradiol.

**Figure S12.** Predicted and observed LNG concentration (Kuhnz et al) vs time plot after 21 days of oral QD dosing of 0.125 mg of levonorgestrel + 0.03 mg of ethinyl estradiol.

Figure S13. Simulated and observed (solid circles; Carten et al) LNG pharmacokinetic profile (dark grey) and SHBG levels (light grey; Kunhz et al) following multiple doses of the combined oral contraceptive.

**Figure S14.** Predicted LNG concentration vs time plot of 0.25 mg of levonorgestrel + 0.05 mg of ethinyl estradiol in the absence (black) or presence (red) of 600 mg QD of carbamazepine.

**Figure S15.** Predicted LNG concentration vs time plot of 0.75 mg of levonorgestrel in the absence (black) or presence (red) of 600 mg of efavirenz.

**Figure S16.** Predicted LNG concentration vs time plot of 0.30 mg of levonorgestrel in the absence (black) or presence (red) of 600 mg of rifampin.

Figure S17. LNG concentration vs time plot of simulated and observed (Natavio et al) LNG for obese women (BMI 30 to 39.9) after receiving a single oral dose of 1.5 mg of LNG.

Figure S18. LNG concentration vs time plot of simulated and observed (Praditpan et al) for women with normal BMI in fed conditions after a single dose of 1.5 mg of LNG.

**Figure S19.** Predicted LNG concentration vs time plot of 0.30 mg of levonorgestrel + 0.03 mg of ethinyl estradiol in the absence (black) or presence (red) of 100 mg QD of itraconazole.

**Figure S20.** Predicted LNG concentration vs time plot of 0.30 mg of levonorgestrel + 0.03 mg of ethinyl estradiol in the absence (black) or presence (red) of 200 mg QD of itraconazole.

**Figure S21.** Predicted LNG concentration vs time plot of 0.30 mg of levonorgestrel + 0.03 mg of ethinyl estradiol in the absence (black) or presence (red) of 400 mg QD of ketoconazole.

| Formulation | AUCR |
| --- | --- |
| ITRA (100 mg) / LNG + EE | 1.27 |
| / LNG-only | 1.49 |
| ITRA (200 mg) / LNG + EE | 1.35 |
| / LNG-only | 1.59 |
| CLR (250 mg BID) / LNG + EE | 1.16 |
| / LNG-only | 1.19 |
| CLR (500 mg BID) / LNG + EE | 1.26 |
| / LNG-only | 1.37 |

Table S9. Summary of LNG AUCR (inhibited vs uninhibited ratios for AUC_0-t_ of PBPK model predictions for the itraconazole (ITRA) (100 or 200 mg QD) and LNG (0.03 mg) with and without EE (0.03 mg) DDI simulations as well as the clarithromycin (CLR) (250 or 500 mg QD) and LNG (0.03 mg) with and without EE (0.03 mg) DDI simulations.

| Pharmacokinetic Parameter | Ratio (Pred/Obs) fasted | Ratio (Pred/Obs) fed |
| --- | --- | --- |
| AUC (ng*h/mL) | **0.95** | **0.82** |
| C_max_ (ng/mL) | **0.88** | **0.93** |

Table S10. Summary of predicted vs observed (Natavio et al) LNG PK parameters, under fed and fasting conditions, for the 1.5 mg PO levonorgestrel simulations.

| Pharmacokinetic Parameter | Observed Normal BMI | Predicted Normal BMI | Observed BMI > 30 | Predicted BMI > 30 |
| --- | --- | --- | --- | --- |
| Cl (L/h) | **4.48 ± 1.19** | **4.39** | **8.51 ± 3.71** | **8.93** |

Table S11. Summary of predicted vs observed (Natavio et al) LNG clearance in normal BMI and obese patients for the 1.5 mg PO levonorgestrel simulations.

Figure S11. Predicted and observed (Kuhnz et al) LNG concentration vs time plot after a single oral dose of 0.125 mg of levonorgestrel + 0.03 mg of ethinyl estradiol.

**Figure S12.** Predicted and observed LNG concentration (Kuhnz et al) vs time plot after 21 days of oral QD dosing of 0.125 mg of levonorgestrel + 0.03 mg of ethinyl estradiol

| 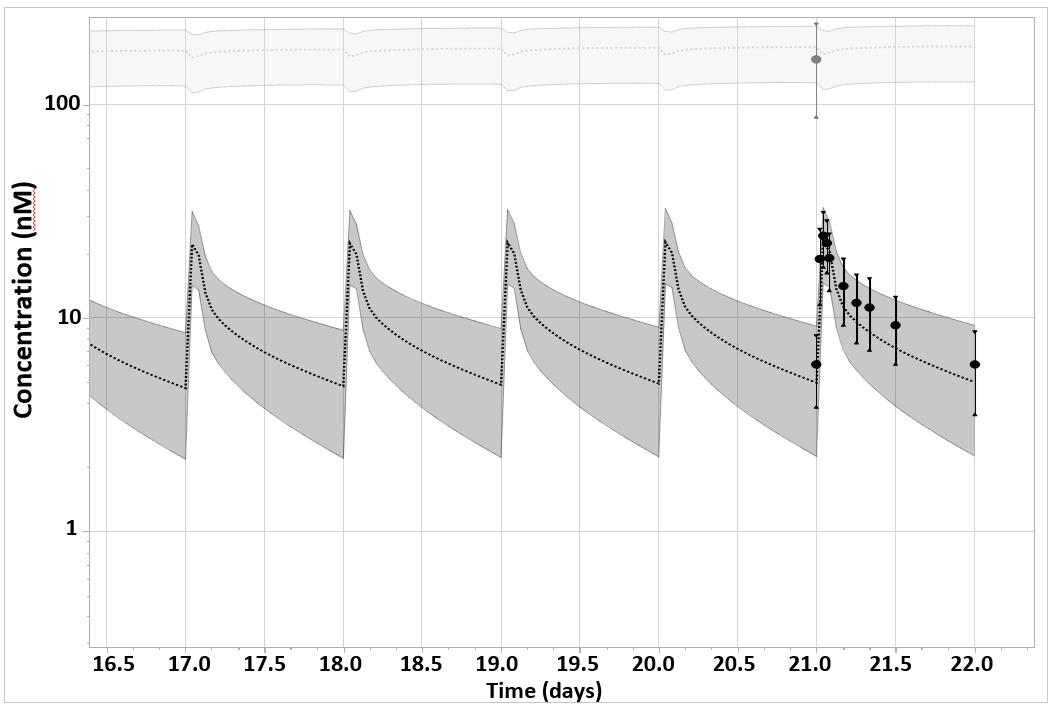 |
| --- |

Figure S13. Simulated and observed (solid circles; Carten et al) LNG pharmacokinetic profile (dark grey) and SHBG levels (light grey; Kunhz et al) following multiple doses of the combined oral contraceptive.

Figure S14. Predicted LNG concentration vs time plot of 0.25 mg of levonorgestrel + 0.05 mg of ethinyl estradiol in the absence (black) or presence (red) of 600 mg QD of carbamazepine.

Figure S15. Predicted LNG concentration vs time plot of 0.75 mg of levonorgestrel in the absence (black) or presence (red) of 600 mg of efavirenz.

Figure S16. Predicted LNG concentration vs time plot of 0.30 mg of levonorgestrel in the absence (black) or presence (red) of 600 mg of rifampin.

Figure S17. LNG concentration vs time plot of simulated and observed (Natavio et al) LNG for obese women (BMI 30 to 39.9) after receiving a single oral dose of 1.5 mg of LNG.

Figure S18. LNG concentration vs time plot of simulated and observed (Praditpan et al) for women with normal BMI in fed conditions after a single dose of 1.5 mg of LNG.

Figure S19. Predicted LNG concentration vs time plot of 0.30 mg of levonorgestrel + 0.03 mg of ethinyl estradiol in the absence (black) or presence (red) of 100 mg QD of itraconazole.

Figure S20. Predicted LNG concentration vs time plot of 0.30 mg of levonorgestrel + 0.03 mg of ethinyl estradiol in the absence (black) or presence (red) of 200 mg QD of itraconazole.

Figure S21. Predicted LNG concentration vs time plot of 0.30 mg of levonorgestrel + 0.03 mg of ethinyl estradiol in the absence (black) or presence (red) of 400 mg QD of ketoconazole.

**Supplemental References in the order as they appear:**

Gérardin A, Dubois JP, Moppert J, Geller L. Absolute bioavailability of carbamazepine after oral administration of a 2% syrup. Epilepsia. 31(3):334-8. (1990).

Zhang X, Lionberger RA, Davit BM, Yu LX. Utility of physiologically based absorption modeling in implementing Quality by Design in drug development. AAPS J. (1):59-71. (2011).

Kohlmann P, Stillhart C, Kuentz M, Parrott N. Investigating Oral Absorption of Carbamazepine in Pediatric Populations. AAPS J. 19(6):1864-1877. (2017).

Vermet H, et. al. Evaluation of Normalization Methods To Predict CYP3A4 Induction in Six Fully Characterized Cryopreserved Human Hepatocyte Preparations and HepaRG Cells. Drug Metab Dispos. 44(1):50-60. (2016).

Backman JT, Olkkola KT, Ojala M, Laaksovirta H, Neuvonen PJ. Concentrations and effects of oral midazolam are greatly reduced in patients treated with carbamazepine or phenytoin. Epilepsia. 37(3):253-7. (1996).

Hanke, N. et. al. *PBPK Models for CYP3A4 and P-gp DDI Prediction: A Modeling Network of Rifampicin, Itraconazole, Clarithromycin, Midazolam, Alfentanil, and Digoxin.* CPT: Pharmacometrics & Systems Pharmacology, 7(10), 647-659. (2018). doi:10.1002/psp4.12343

Crawford P, Chadwick DJ, Martin C, et al. The interaction of phenytoin and carbamazepine with combined oral contraceptive steroids. Br J Clin Pharmacol. 30(6): 892–896. (1990).

Limpongsanurak, S., Jenkins, N., & Fotherby, K. Effect of contraceptive steroids on serum levels of sex hormone binding globulin and caeruloplasmin. *Current Medical Research and Opinion,* *7*(3), 185-191. (1981). doi:10.1185/03007998109114261

Bayer AG. Hormonal contraceptive agents database (HCAD). <<https://github.com/Open-Systems-Pharmacology/Hormonal-Contraceptive-Agents-Datasets/blob/master/ocp_dataset.xlsx>> (2018)

Falcão, A., Vaz-Da-Silva, M., Gama, H., Nunes, T., Almeida, L., Soares-Da-Silva, P. *Effect of eslicarbazepine acetate on the pharmacokinetics of a combined ethinylestradiol/levonorgestrel oral contraceptive in healthy women*. Epilepsy Research, 105(3), 368-376. (2013). doi:10.1016/j.eplepsyres.2013.02.020

Kuhnz, W., Blode, H., Mahler, M. *Systemic availability of levonorgestrel after single oral administration of a norgestimate-containing combination oral contraceptive to 12 young women*. Contraception, 49(3), 255-263. (1994). doi:10.1016/0010-7824(94)90043-4

Kook, K., Gabelnick, H., Duncan, G. *Pharmacokinetics of levonorgestrel 0.75 mg tablets.* Contraception, 66(1), 73-76. (2002). doi:10.1016/s0010-7824(02)00321-9

He, C. et. al. *Comparative cross-over pharmacokinetic study on two types of postcoital contraceptive tablets containing levonorgestrel.* Contraception, 41(5), 557-567. (1990). doi:10.1016/0010-7824(90)90064-3

Tremblay, D., Gainer, E., Ulmann, A. *The pharmacokinetics of 750 μg levonorgestrel following administration of one single dose or two doses at 12- or 24-h interval.* Contraception, 64(6), 327-331. (2001). doi:10.1016/s0010-7824(01)00276-1

Carten ML, et al. Pharmacokinetic Interactions between the Hormonal Emergency Contraception, Levonorgestrel (Plan B), and Efavirenz. Infectious Diseases in Obstetrics and Gynecology, 2012, 1–4. (2012) doi:10.1155/2012/137192.

Natavio M, et al. Pharmacokinetics of the 1.5 Mg Levonorgestrel Emergency Contraceptive in Women with Normal, Obese and Extremely Obese Body Mass Index. Contraception, 99(5), 306–311. (2019) doi:10.1016/j.c

Praditpan, P., Hamouie, A., Basaraba, C. N., Nandakumar, R., Cremers, S., Davis, A. R., & Westhoff, C. L. Pharmacokinetics of levonorgestrel and ulipristal acetate emergency contraception in women with normal and obese body mass index. *Contraception,* *95*(5), 464-469. (2017). doi:10.1016/j.contraception.2017.01.004
